# 

**DOI:** 10.1038/sj.bjc.6600405

**Published:** 2002-01-02


					not significantly different across response types (complete, partial, or no
response - p=0.80) and was not predictive for time to symptomatic
progression. Response rate was dependent on site of metastases (p=0.002)
with bone having the greatest likelihood of response (96% CR or PR).
Patients with bone metastases received less total dose (p=0.001), less
biological effective dose (p=0.001), and had a significantly longer time to
progression than other sites (hazard ratio 0.4 - 95% CI 0.2-0.7; p=0.004).
Primary interferon treatment (i.e. treatment of the primary tumour with
interferon only, without surgery or radiotherapy) was also associated with a
shorter time to progression (hazard ratio 4.6 - 95% CI 1.5-14.1, p=0.007). On
removal of these criteria, brain metastases became a significant predictor of
progression time with a hazard ratio of 2.5 (95% CI 1.0 - 5.9; p=0.05)
showing a increased risk of progression with brain metastases compared to
metastases at other sites. Patients presenting with spinal cord compression
had a particularly poor response rate (50%). Similar results were found from
the sensitivity analysis.
CONCLUSION: Despite the widespread assumption that renal cell
carcinoma is radioresistant, response rates to palliative radiotherapy are high.
Higher BED does not appear from our data to be a predictor of symptomatic
improvement or duration of response. Palliation of bone pain seems to be
particularly durable compared to the palliation of symptoms at other sites of
metastases. A trend for shorter duration of the palliative effect of whole brain
radiotherapy is noted. Wherever possible, patients presenting with spinal cord
compression are more appropriately treated with surgery in the first instance.
3.8
OPTIMAL RADIOTHERAPY DOSE FOR METASTATIC SPINAL
CANAL COMPRESSION.
*P J Hoskin, A Grover, R Bhana; Mount Vernon Cancer Centre,
Mount Vernon Hospital, Northwood, Middlesex HA6 2RN, UK.
Optimal radiotherapy dose fractionation for spinal cord compression has not
been defined. This retrospective study has compared patients treated in a
single centre receiving either 1 or 2 fractions of radiotherapy or a fractionated
course of more prolonged treatment for spinal cord compression.
A total of 102 consecutive patients have been analysed, treated between
January 1999 and June 2001. Sixty patients were male with a median age of
68 (range 32 to 90 years). The most common primary sites were breast
(28%), prostate (28%) and lung (20%). The median duration of symptoms
prior to diagnosis was 3 weeks (range 0 to 40); diagnosis within 24 hours of
symptoms was achieved in only 13% and within one week in 29%. The
diagnosis was confirmed on MRI in 93 patients (95%); the site of
compression was dorsal spine (58%), lumbosacral (33%) and cervical cord
(9%). Multiple levels of compression were present in 34 patients (33%). In
93 patients (91%) there were other sites of metastatic disease the most
common of these being other bone metastasis.
51% were fully ambulant at diagnosis and 8% had complete paraplegia, the
remainder having paraparesis with impaired mobility. At two months after
RT the number of patients fully ambulant without significant neurological
signs increased from 51% to 71% but the number of patients with complete
paraplegia remained unchanged at 8%. A reduction in the incidence of
impaired bladder function from 31% at presentation to 14% over the same
time period was seen. Pain scores were derived for all patients on a simple
retrospective 4 point scale. At presentation 59% recorded severe pain ( grade
III) and 30% moderate pain (grade II). Only 4% had no pain (grade 0). At
two weeks there was a substantial improvement in pain control with only 8%
recording severe pain.
Patients received a range of radiotherapy dose fractionation schedules the
most common of which were 8Gy in a single fraction (22%) or 20Gy in 5
fractions (64%). In total 24 patients (23.5%) received a single or only 2
fractions (total dose 8 to 12Gy), the remainder receiving 20Gy in 5 fractions
or in one case each 13Gy in 3 fractions, 13Gy in 4 fractions and 40Gy in 20
fractions. Patients receiving 1 or 2 fractions (1/2#) have been compared with
the remainder (multi#). No difference in demographic parameters including
primary tumour type and sites of cord compression was seen. A higher
proportion of patients were paraplegic at presentation in the 1/2# (19%) than
multi# (3%); when comparing outcome at two months from treatment the
number of patients retaining mobility was statistically no different (80%,1/2#
vs 68% multi#) and the proportion remaining paraplegic was unchanged
(20%,1/2# vs 4% multi#). Pain control was also the same in both groups.
In conclusion this series has confirmed that spinal cord compression should
be diagnosed and treated urgently whilst the patient is mobile. Recovery
after established paraplegia is rare. No advantage for protracted courses of
radiotherapy over 1 or 2 fractions has been observed in this retrospective
analysis. This data provides a strong basis for a prospective randomised trial
evaluating dose fractionation in this condition.
4.1
HYPERMETHYLATION OF AN INTRAGENIC CPG ISLAND OF
THE MCJ GENE CORRELATES WITH LOSS OF EXPRESSION
AND DRUG RESISTANCE IN OVARIAN CANCER CELLS
G. Strathdee* and R. Brown, Dept. of Medical Oncology, Cancer Research
UK Beatson Laboratories, Glasgow University G61 1BD
Alterations in DNA methylation probably play a major role in the
development and progression of most, or all, tumour types. Many recent
studies have identified increased methylation within the promoter regions of
genes known to play important roles in cancer and demonstrated that such
promoter hypermethylation was associated with loss of gene expression.
Alteration in DNA methylation outside of promoter regions are also
frequently observed, although the significance of such changes is less clear.
We have previously demonstrated that aberrant promoter hypermethylation is
common in ovarian cancer, targeting numerous genes, including many with
known roles in tumour development (e.g. BRCA1) and resistance to
chemotherapeutic agents (e.g. MLH1).
Recently, the MCJ gene has been identified as a target for aberrant
methylation in ovarian cancer and shown to play a role in sensitivity to
several important anti-cancer drugs, such as cisplatin (Shridhar et al 2001,
Cancer Res. 61:4258). Analysis of MCJ expression in our cell line models
demonstrated that expression of the gene was lost in 8/10 cisplatin-resistant
derivatives of the ovarian carcinoma cell line A2780. Furthermore, treatment
of two of the resistant cell lines with 5-azacytidine, which inhibits DNA
methyltransferase activity, resulted in re-expression of MCJ, suggesting that
loss of expression may be due to increased methylation. Although the MCJ
gene does not contain a classic CpG island within its promoter a previous
report suggested that methylation of one particular CpG site within the
promoter correlated with loss of expression. However, bisulfite sequencing
analysis of this region, in the A2780 derivatives, identified no correlation
between methylation of this, or other proximal CpG sites and gene
expression. However, a CpG island was identified beginning within the first
exon of MCJ, 164bp downstream of the transcriptional start site. Bisulfite
sequencing of this region in DNA extracted from normal ovarian tissue
determined that about 50% of clones were densely methylated and about 50%
clones largely unmethylated. Sequencing of expressing cell lines revealed a
pattern of methylation similar to normal DNA, whereas the cisplatin-
resistant, non-expressing, cell lines exhibited dense methylation of all clones.
5-azacytidine treatment of one of the non-expressing cell lines, which
resulted in re-expression of MCJ, gave a methylation pattern similar to the
MCJ expressing cell lines. These results suggest that methylation of the
intragenic CpG island of MCJ may be responsible for loss of gene
expression. Furthermore, sequencing of this region in ovarian tumour
samples identified a subset of tumour (30%) which, similar to the non-
expressing ovarian cell lines, exhibited high levels of methylation of all
clones. This raises the possibility that increased methylation of MCJ could
play a role in controlling MCJ expression, and consequently in determining
drug sensitivity, in ovarian cancer.
4.2
EXPRESSION OF SMAC/DIABLO IN OVARIAN CARCINOMA
CELLS INDUCES APOPTOSIS PREDOMINANTLY VIA A
CASPASE-9-MEDIATED PATHWAY AND ENHANCES
SENSITIVITY TO CISPLATIN AND PACLITAXEL
I.A. McNeish*, S. Bell, T. McKay, T. Tenev, V. Stoll, N.R. Lemoine
Cancer Research UK Molecular Oncology Unit, Imperial College School of
Medicine, Hammersmith Hospital, LONDON W12 0HS
A key step in the execution of apoptosis is the activation of caspases, one
pathway of which involves the release of cytochrome c from mitochondria,
which then binds to Apaf-1 leading to the activation of caspase-9. This, in
turn, activates downstream execution caspases. Recently, a second
mitochondrial activator of caspases, known as Smac or DIABLO, was
described, which is proposed to function by inhibiting the anti-apoptotic
Oral Presentations
S20
British Journal of Cancer (2002) 86(Suppl 1), S13 - S33  2002 Cancer Research UK
protein XIAP (X-linked Inhibitor of Apoptosis Protein). The suggested
mechanism is an interaction between the N-terminal region of Smac and the
BIR3 domain of XIAP, although there is also evidence that a Smac mutant
that lacks the N-terminal amino acids still remains capable of potentiating
apoptosis. Recently, it was shown that release of Smac from mitochondria is
involved in dexamethasone-induced apoptosis in multiple myeloma cells and
may a key link between the two pathways of caspase activation.
We have constructed Ad CMV-Smac, a recombinant adenovirus encoding
Smac/DIABLO. We demonstrate for the first time that delivery of the Smac
gene to malignant cells can induce apoptosis: transfection of ovarian
carcinoma cells with Ad CMV-Smac at multiplicities of infection of 3 - 30
pfu/cell leads to increasing apoptosis in a dose-dependent manner. Western
blot analysis confirms that Smac-induced apoptosis proceeds via a caspase-9
dependent pathway, which can be partially inhibited by the caspase-9
inhibitor zLEHD-fmk and by over-expression of XIAP. At later time points,
however, there is also caspase-8 activation, which suggests that Smac may be
able to activate multiple apoptotic pathways. We also show that Ad CMV-
Smac can combine with other pro-apoptotic factors, such as cisplatin,
paclitaxel and pro-caspase-3, to produce greater levels of apoptosis in
transfected cells.
Abnormalities in the cytochrome c/Apaf-1 pathway of caspase activation
have been found in ovarian carcinoma cells. Furthermore, there is evidence
that XIAP may be intimately linked to chemotherapy resistance in ovarian
cancer and that down-regulation of XIAP can induce apoptosis in
chemoresistant ovarian cancer cells. In light of this, we suggest that up-
regulation of Smac activity may have genuine potential in the treatment of
ovarian cancer.
4.3



-GALACTOSIDE BINDING PROTEIN (


GBP) SELECTIVELY
REDUCES THE PROLIFERATIVE CAPACITY OF PRIMARY CML
CELLS AND EVADES P210BCR-ABL
MEDIATED RESISTANCE TO
APOPTOSIS; A ROLE FOR A NATURAL CYTOKINE IN TUMOUR
REGULATION.
Spellacy N1
, McElwaine S1
, Mallucci L2
, Wells V2
, Lawler M1 1
Dept.
Haematology/Oncology, St.James Hospital and TCD, 2
Cell Growth
Regulation Laboratory, Kings College London
Chronic myelogenous leukaemia (CML) is characterised at the molecular
level by a (9;22) translocation which places the abl oncogene under the
control of the bcr promoter generating a fusion protein (p210) with enhanced
tyrosine kinase activity. CML cells are inherently resistant to apoptosis. The
negative regulatory cytokine -galactoside binding protein (-GBP) induces
cell cycle arrest at the late S/G2 threshold. Negation of this block via anti-
GBP antibodies allows normal cells to resume growth but neoplastic cells
undergo apoptosis. Preliminary results indicate that lymphomas, mammary
cell cancers and certain leukaemic cells undergo apoptosis following
treatment with -GBP at nanomolar concentrations, suggesting that GBP
may offer a novel and selective means of controlling neoplastic growth. We
have examined the effect of GBP on CML cell lines (K562, BV173,
LAMA84, KYO-1) and have demonstrated apoptosis in 30-50% of the cells
via TUNEL assays and Annexin V staining at 48 hours. Dual staining with
propidium iodide, indicating cell cycle distribution, showed that at 24 hours
cells exposed to GBP had been arrested in S phase. Thus GBP blocks the
cell cycle in CML prior to activating cell death. These results prompted
analysis of the effect of -GBP on primary hemopoeitic progenitor cells
(HPC's) from bone marrow donors and CML patients using in vitro colony
forming assays. Treatment of HPC's from normal BM donors (n=22) with
400ng/ml GBP did not significantly reduce CFU-GM number (median
inhibition 0 -17%). By contrast treatment of HPC's from CML patients at
diagnosis (n=13) resulted in a significant decrease in colony number (median
inhibition 49-63%, p <0.005). Thus GBP has a significant effect on the
proliferative ability of leukemic progenitors while sparing normal
hemopoeitic progenitor cells. Treatment of CML cells with GBP also
resulted in a significant decrease in the levels of the p210BCR-ABL
protein after
40-44 hours incubation and this downregulation preceded the appearance of
significant numbers of apoptotic cells. Quantitation of bcr-abl mRNA levels
by real time PCR using TaqMan technology indicated that mRNA levels are
unchanged following treatment with GBP indicating that P210
downregulation occurs at the post transcriptional level. p210 downregulation
by GBP is caspase-3 and proteasome independent. Thus two critical events,
the prior introduction of a cell cycle block, allied to the subsequent down-
regulation of p210BCR-ABL
may both be required for apoptosis induction by
GBP in CML. The selective anti-proliferative and pro-apoptotic effect of
GBP on leukemic HPC's and its ability to downregulate p210BCR-ABL
may
offer a new method to selectively induce apoptosis in CML cells while
sparing normal hemopoietic progenitors. This may be useful in purging
marrow in the context of autografting for CML or as an in vivo treatment,
either alone or in combination with other chemotherapeutic agents.
4.4
IDENTIFICATION OF 5-FU INDUCIBLE GENES IN TUMOUR
CELL LINES BY cDNA MIRCROARRAY
Pamela J Maxwell*
, Daniel B Longley, John Boyer, Tariq Latif, Wendy
Allen, Maria Lynch, D Paul Harkin, and Patrick G Johnston,
Department of Oncology, Cancer Research Centre, Queen's University
Belfast, Belfast, N. Ireland.
Thymidylate synthase (TS) is a critical chemotherapeutic target for
fluoropyrimidines such as 5-fluorouracil (5-FU) and antifolates such as
tomudex (TDX). The major limitations to TS-directed therapies are acquired
or inherent resistance of tumour cells to these agents. Previous studies have
demonstrated that TS expression levels are a key determinant of sensitivity of
tumour cells to 5-FU and TDX. In order to identify more potential markers of
response to TS-directed therapy, we have used DNA microarray technology
to identify genes that are induced by 5-FU treatment in the MCF-7 breast
cancer cell line. Of 2,400 genes analysed 30 were up-regulated by >8-fold. Of
10 highly up-regulated genes identified by the DNA microarray, only 6 were
consistently found to be up-regulated in a dose and time dependent manner
by Northern blot analysis. Consistently up-regulated genes included
spermine/spermidine acetyl transferase (SSAT), annexin II, MAT8 and
thymosin -10. Analysis of other cell lines treated with 5-FU revealed that
the induction of SSAT, annexin II, MAT8 and thymosin -10 by 5-FU was
not cell line specific. Furthermore, analysis of MCF-7 cells treated with TDX
and oxaliplatin indicated that the expression of SSAT, annexin II, MAT8 and
thymosin -10 could be induced by both TS-specific and non-specific
therapies. In addition, our results have shown that the expression of MAT-8
and thymosin -10 were reduced significantly in a TDX resistant MCF-7 cell
line compared to the parental line.
Our results suggest that SSAT, annexin II MAT8 and thymosin -10 may be
involved in mediating the response of tumour cells to chemotherapy.
Furthermore, our analysis demonstrates the potential of DNA microarrays to
identify novel predictive markers and/or therapeutic targets.
4.5
CYCLO-OXYGENASE INHIBITORS REDUCE METASTASIS, AND
SERUM VEGF FOLLOWING EXCISION OF A PRIMARY
TUMOUR.
G Roche-Nagle*, J Harmey, E Connolly, D Bouchier-Hayes. Department of
Surgery, Beaumont Hospital, Dublin 9.
Cyclo-oxygenase-2 (COX-2) expression is increased in breast cancer and
surgery has been shown to increase the growth of metastatic tumours. The
aim of our experiment was to investigate the effect of selective COX-2
inhibition on tumour metastases in an experimental excision model of breast
cancer.50000 4T1 mammary carcinoma cells were injected into the
mammary fat pad of 9 week old female BALB/c mice .On day 14 post
injection (mean tumour size= 8.0+/-0.4 mm) the primary tumours were
excised and the mice were randomised into 2 groups (n=7/group). One group
received daily intra peritoneal (IP) injections of the selective COX-2
inhibitor, SC-236, the second group only received drug vehicle. Animals
were sacrificed 14 days post excision of primary. At harvest there was a
significant reduction in the number of lung metastases and serum VEGF
levels between the SC-236 and control group. We also found a reduction in
microvessel density and an increase in the apoptotic index.
Oral Presentations
S21
 2002 Cancer Research UK British Journal of Cancer (2002) 86(Suppl 1), S13 - S33
Oral Presentations
S22
British Journal of Cancer (2002) 86(Suppl 1), S13 - S33  2002 Cancer Research UK
determined by the collection-rate method (2). Results show that the parent
K562 and MCF cells exhibit significantly lower cytoplasmic conductivities of
0.026Sm-1
and 0.025Sm-1
, respectively than their drug resistant cells:- 0.3
Sm-1
and 0.45Sm-1
, K562AR and MCF-7mdr, respectively. Furthermore,
treatment of resistant cells with the Pgp specific MDR inhibitor XR 9576
showed a significant lowering of the internal conductivity to a value similar
to that of the parental cells. A decrease in membrane permittivity was
observed after treating K562AR with the modulator (from 10 to 6.9).
In conclusion, We propose that this method offers great potential for the rapid
assessment of the mechanisms of cancer drug action in vitro.as these data
indicate that the electrical character of the cell is altered in MDR, but
following modulation therapy, MDR cells achieve a similar profile to drug
sensitive cells.
(1) Gascoyne, P.R.C. Wang, X. Huang, Y. Becker, F. 1997, IEEE
Transactions on industry applications, Vol. 33(3): 670. (2) Gascoyne, P.R.C.
Pethig, R. Burt, J., Becker, F. 1993, Biochemica et Biophysica Acta, Vol.
1149:119. (3) Vyuvegula, B. et al, 1988, Cancer Chemotherapy and
Pharmacology, Vol. 22:163.
5.1
UPDATED RESULTS OF A RANDOMISED CONTROLLED TRIAL
OF NEOADJUVANT CISPLATIN (C), METHOTREXATE (M) AND
VINBLASTINE (V) CHEMOTHERAPY FOR MUSCLE-INVASIVE
BLADDER CANCER.
JT Roberts* on behalf of the International collaboration of trialists of the
MRC advanced bladder cancer group, EORTC GU group, NCIC clinical
trials group, CUETO group, Australian bladder cancer study group,
Finnbladder and Norwegian bladder cancer study group. c
/o MRC Clinical
Trials Unit, 222 Euston Road, London NW1 2DA, UK.
An international multi-centre randomised controlled trial was conducted
comparing whether the addition of neoadjuvant CMV to radical surgery or
radiotherapy for patients with muscle-invasive transitional cell carcinoma of
the bladder would improve survival. Eligible patients had histologically
confirmed T2(G3), T3, T4a, N0/NX, M0 transitional cell carcinoma of the
bladder and were judged suitable for curative treatment. CMV was given as 3
cycles consisting of 30mg/m2
M & 4mg/m2
V on days 1,8 and 100mg/m2
C
on day 2 of each cycle. From November 1989 to July 1995 a total of 976
patients were entered by 106 centres in 20 countries, 491 randomised to
CMV and 485 to no CMV. 36% of patients were <60 years, 88% male, 69%
WHO PS 0, 58% T3, 65% N0 and 88% G3. First results with a median
follow-up of 4 years have been reported1
in 1999 which showed a statistically
non-significant 15% decrease in the risk of death after CMV (Hazard ratio
0.85 [95% CI 0.71-1.02] p=0.075). Currently a total of 559 patients have died
and a final analysis is planned early 2002. At this time the median follow-up
will be approximately 7 years. This is the largest trial making this comparison
and the mature data will enable us to examine whether there is any evidence
of CMV having a long-term effect on the main outcome of overall survival
and other endpoints.
1
International collaboration of trialists. Neoadjuvant cisplatin, methotrexate,
and vinblastine chemotherapy for muscle invasive bladder cancer: a
randomised controlled trial. The Lancet 1999, 354, p533-40.
5.2
THE EMI STUDY: A REGIONAL FEASIBILITY STUDY FOR A
RANDOMISED TRIAL OF ADJUVANT CHEMOTHERAPY
FOLLOWING DEFINITIVE TREATMENT FOR TRANSITIONAL
CELL CANCER (TCC) OF THE BLADDER.
*MG Leahy1
, J Brown2
, WG Jones3
, J Kelly4
, DE Neal4
, S Prescott5
, T
Roberts6
, P Selby7
1
University of Leeds, School of Medicine. 2
Northern and Yorkshire Clinical
Trials and Research Unit. 3
Leeds Cancer Centre. 4
University of Newcastle. 5
Leeds Teaching Hospitals NHS Trust. 6
Northern Centre for Cancer
Treatment. 7
Cancer Research UK Clinical Centre in Leeds.
Introduction: Cancer networks have the potential to make a powerful
contribution to cancer research through collaborative studies. We conducted
a feasibility study in the Northern and Yorkshire Region that attempted to
recruit all eligible patients (pts.) into a randomised trial of adjuvant
chemotherapy following radical local treatment for muscle invasive TCC of
the bladder.
Patients and Methods: The protocol was approved by Northern and
Yorkshire Multicentre Research Ethics Committee. The target was to
randomise 60 pts. within 2 years. Consenting pts. were registered at
diagnosis. Following primary therapy (cystectectomy [CYST] or radical
radiotherapy [RRT]), pts. were assessed for fitness to start 3 cycles of MVAC
chemotherapy within 12 weeks. If suitable, they were given further
information and randomised if they consented.
Result: The trial was activated in 20 hospitals with enthusiastic support. 354
pts. were registered. 21% were not suitable for radical primary therapy due to
metastatic disease or co-morbidity. Of the remainder, 50% had CYST and
50% had RRT. After CYST/RRT, 67% / 81% were medically unfit for
chemotherapy. Of the 15% eligible for randomisation, 75% declined. The
final number of patients randomised was 6.
Conclusion: This study demonstrates that population-based cancer research
within the NHS is feasible. In the patient group studied there was a higher
incidence of co-morbidity than expected and many pts. declined
randomisation. This experience is relevant to the development of the new
National Cancer Research Network. The difficulty in recruitment in this area
supports the recent decision by co-operative groups to collaborate in a
international intergroup study to address the issue of adjuvant chemotherapy
for bladder cancer.
5.3
MICROARRAY GENERATED ONCOGENE COPY NUMBER
PROFILING IN HUMAN BLADDER CANCER.
A. McGoldrick1
, A. McCann*1
, J. Fitzpatrick 3
, J. Smith 3
and P. A.
Dervan 1, 2
,
1
Department of Pathology, Conway Institute of Biomolecular and
Biomedical Research, University College Dublin, UCD, Belfield, Dublin
4, Ireland.
2
Department of Pathology, Mater Misericordiae Hospital, Eccles Street,
Dublin 7, Ireland.
3
Department of Surgery, University College Dublin (UCD) and Mater
Hospital, Eccles Street, Dublin 2 Ireland.
Using the Vysis GenoSensor Micro array System and the AmpliconI
oncogene specific chip, we have screened 59 oncogenes for altered copy
number in a cohort of 30 bladder patient samples consisting of 14 paired
and 2 unpaired transitional cell carcinomas (TCC's). Following DNA
extraction, nick translation and hybridisation, amplification profiles for
each of the 59 arrayed oncogenes were generated. Using a stringent cut
off value of 1.75, seven tumour samples showed normal copy number at
all loci, while 9 tumours showed increased copy number of 1 (n=7) or
multiple loci (n=2) involving 12 out of the 59 arrayed oncogenes when
compared to paired normal samples. The genes identified were MDM2
(12q14.3), c-erbB-2 (17q), FGR (1p36), PIK3CA (3q26.3), ZNF127
(20q), AR (Xq) and JUNB (19p13.2). The c-MET (7q21) locus was the
most commonly altered gene, with 3/16 (19%) tumours displaying
increased copy number. The findings with c-MET are novel and
significant for a number of reasons. Firstly, it functions as the receptor
for the hepatocyte growth factor/ scatter factor (HGF/SF); a growth
factor known to be a marker of disease stage and poor outcome in
bladder cancer (Gohji, K., et al., 2000). Secondly, stimulation of c-met
by its ligand HGF/SF leads to a whole range of biological downstream
affects including scattering, angiogenesis, proliferation enhanced cell
motility and invasion (Reviewed Maulik, G., et al., 2002). Finally, due to
data already indicating the efficacy of receptor specific inhibitors to the
c-kit and PDGFR receptor tyrosine kinases in gastrointestinal stromal
tumours and NCLC tumours respectively, the possibility that c-MET
may also be a suitable target for such antineoplastic therapy is clinically
significant.
References:-
Gohji, K., Nomi, M., Niitani, Y., Kitazawa, S., Fujii, A., Katsuoka, Y.,
and Nakajima, M. 2000, J. Clin. Oncol., 18, 2963.
Maulik G, Shrikhande A, Kijima T, Ma PC, Morrison PT, and Salgia R,
2002, Cytokine Growth Factor Rev 13, 41.
Oral Presentations
S23
 2002 Cancer Research UK British Journal of Cancer (2002) 86(Suppl 1), S13 - S33
5
5.
.4
4
I
IN
NF
FL
LU
UE
EN
NC
CE
E O
OF
F C
CY
YT
TO
OK
KI
IN
NE
E P
PO
OL
LY
YM
MO
OR
RP
PH
HI
IS
SM
MS
S O
ON
N
S
SU
US
SC
CE
EP
PT
TI
IB
BI
IL
LI
IT
TY
Y T
TO
O A
AN
ND
D/
/O
OR
R P
PR
RO
OG
GN
NO
OS
SI
IS
S I
IN
N P
PR
RO
OS
ST
TA
AT
TE
E
C
CA
AN
NC
CE
ER
R
*SL McCarron1
, S Edwards2
, PR Evans1
, A Dowe2
, A Ardern-Jones2
, C
Southgate2
, D Dearnaley2
, The CRC/BPG UK Familial Prostate Cancer Study
Collaborators, 3
DF Easton, R Eeles2
, WM Howell1
, 1
Histocompatibility and
Immunogenetics Laboratory, Southampton University Hospitals,
Southampton, 2
Institute of Cancer Research, Royal Marsden Hospital, Sutton,
3
CRC Genetic Epidemiology Unit, Strangeways Research Laboratory,
Cambridge, UK
Prostate cancer (PC) is one of the leading causes of male deaths in the
western hemisphere with approximately 134,000 new cases and 56,000
deaths occurring annually in the EU. Polymorphisms in immune response
genes may influence susceptibility to and/or prognosis in sporadic cases of
PC, resulting from inter-individual differences in the anti-tumour immune
response.
In this study, 250 PC patients and 256 cancer-free controls were genotypes by
ARMS-PCR for 5 polymorphisms: Interleukin (IL) -1 (-511, A/G), IL-8 (-
251, A/T), IL-10 (-1082, A/G), Tumour Necrosis Factor- (-308, A/G)
and Vascular Endothelial Growth Factor (VEGF), (-1154, A/G). P
Pa
at
ti
ie
en
nt
t
c
co
on
nt
tr
ro
ol
l g
ge
en
no
ot
ty
yp
pe
e c
co
om
mp
pa
ar
ri
is
so
on
ns
s r
re
ev
ve
ea
al
le
ed
d t
th
ha
at
t I
IL
L-
-8
8 A
AA
A (
(h
hi
ig
gh
h p
pr
ro
od
du
uc
ce
er
r)
) a
an
nd
d
V
VE
EG
GF
F A
AA
A (
(l
lo
ow
w p
pr
ro
od
du
uc
ce
er
r)
) g
ge
en
no
ot
ty
yp
pe
es
s w
we
er
re
e s
si
ig
gn
ni
if
fi
ic
ca
an
nt
tl
ly
y d
de
ec
cr
re
ea
as
se
ed
d i
in
n t
th
he
e
p
pa
at
ti
ie
en
nt
ts
s w
wh
he
en
n c
co
om
mp
pa
ar
re
ed
d t
to
o t
th
he
e c
co
on
nt
tr
ro
ol
ls
s,
, (
(2
23
3.
.9
9%
% v
v 3
32
2.
.3
3%
%;
; O
OR
R=
=0
0.
.6
66
6;
; p
p=
=0
0.
.0
04
4)
)
a
an
nd
d (
(6
6.
.3
3%
% v
v 1
12
2.
.9
9%
%;
; O
OR
R=
=0
0.
.4
45
5;
; p
p=
=0
0.
.0
01
1)
) r
re
es
sp
pe
ec
ct
ti
iv
ve
el
ly
y.
. I
IL
L-
-1
10
0 A
AA
A (
(l
lo
ow
w
p
pr
ro
od
du
uc
ce
er
r)
) g
ge
en
no
ot
ty
yp
pe
e w
wa
as
s s
si
ig
gn
ni
if
fi
ic
ca
an
nt
tl
ly
y i
in
nc
cr
re
ea
as
se
ed
d i
in
n t
th
he
e p
pa
at
ti
ie
en
nt
ts
s w
wh
he
en
n
c
co
om
mp
pa
ar
re
ed
d t
to
o t
th
he
e c
co
on
nt
tr
ro
ol
ls
s,
, (
(3
31
1.
.6
6%
% v
v 1
17
7.
.1
1%
%;
; O
OR
R=
=2
2.
.2
24
4;
; p
p=
=0
0.
.0
00
01
1)
).
. W
Wi
it
th
hi
in
n t
th
he
e
P
PC
C g
gr
ro
ou
up
p,
, I
IL
L-
-8
8 g
ge
en
no
ot
ty
yp
pe
e w
wa
as
s a
as
ss
so
oc
ci
ia
at
te
ed
d w
wi
it
th
h p
pr
ro
os
st
ta
at
te
e s
sp
pe
ec
ci
if
fi
ic
c a
an
nt
ti
ig
ge
en
n (
(P
PS
SA
A)
)
l
le
ev
ve
el
l at diagnosis, at a borderline level of significance (
(p
p=
=0
0.
.0
05
5)
).
.
T
Th
he
es
se
e r
re
es
su
ul
lt
ts
s s
su
ug
gg
ge
es
st
t t
th
ha
at
t g
ge
en
no
ot
ty
yp
pe
es
s a
as
ss
so
oc
ci
ia
at
te
ed
d w
wi
it
th
h d
di
if
ff
fe
er
re
en
nt
ti
ia
al
l p
pr
ro
od
du
uc
ct
ti
io
on
n
o
of
f I
IL
L-
-8
8,
, I
IL
L-
-1
10
0 a
an
nd
d V
VE
EG
GF
F a
ar
re
e r
ri
is
sk
k f
fa
ac
ct
to
or
rs
s f
fo
or
r P
PC
C.
. G
Ge
en
no
ot
ty
yp
pe
es
s a
as
ss
so
oc
ci
ia
at
te
ed
d w
wi
it
th
h
h
hi
ig
gh
h I
IL
L-
-1
10
0 a
an
nd
d l
lo
ow
w V
VE
EG
GF
F p
pr
ro
od
du
uc
ct
ti
io
on
n m
ma
ay
y a
ac
ct
t v
vi
ia
a t
th
he
ei
ir
r i
in
nf
fl
lu
ue
en
nc
ce
e o
on
n
a
an
ng
gi
io
og
ge
en
ne
es
si
is
s,
, n
ne
ec
ce
es
ss
sa
ar
ry
y f
fo
or
r m
me
et
ta
as
st
ta
as
si
is
s.
. T
Th
he
er
re
ef
fo
or
re
e p
po
ol
ly
ym
mo
or
rp
ph
hi
is
sm
ms
s in cytokine
genes may influence susceptibility to and prognosis in PC, indicating that a
definitive investigation is required in a larger study group.
5.5
OBCAM, AN IGLON CELL ADHESION MOLECULE FROM 11Q25,
IS FREQUENTLY INACTIVATED IN SPORADIC EPITHELIAL
OVARIAN CANCER
*Sellar GC, Watt KP, Stronach EA, Rabiasz GJ, Miller EP, Massie C, Scott
D, 1
Porteous DJ, Smyth JF and Gabra H. Cancer Research UK Edinburgh
Oncology Unit, Western General Hospital, Edinburgh EH4 2XR, UK.
1
Department of Medical Sciences, Edinburgh University, Molecular
Medicine Centre, Western General Hospital, Edinburgh EH4 2XU, UK.
We present evidence that OBCAM (Opioid Binding Cell Adhesion Molecule)
is a strong candidate tumor suppressor gene (TSG) inactivation of which is a
fundamental event in the development of EOC.
A frequent LOH rate of 51% was observed at the 11q25 marker D11S4085 in
a series of 100 fresh and archive derived EOC normal/tumor pairs in an
analysis using all polymorphic microsatellite markers from the region. In
many cases, LOH demonstrated complete loss of an allele rather than allelic
imbalance, suggesting lack of tumor heterogeneity. This indicated that LOH
at D11S4085 is an early event in epithelial ovarian carcinogenesis.
Bioinformatic analysis mapped D11S4085 within an intron of the OBCAM
gene, a member of the IgLON family of GPI-anchored cell adhesion
molecules. OBCAM may also be involved in signal transduction, and we
have preliminary evidence that this is mediated through the raf/erk pathway.
By quantitative RT-PCR, OBCAM expression is strong in normal human
ovarian surface epithelium, but is extremely weak or undetectable in almost
all primary ovarian tumours and cancer cell lines tested.
Using Methylation Specific PCR, the OBCAM 5' CpG island is methylated
in 81% (13/16) of ovarian cancer cell lines, and somatically methylated in
76% (32/42) of primary EOCs. In contrast, the OBCAM CpG island in
normal ovary is unmethylated. In patients with LOH at D11S4085, i.e. within
OBCAM, the second allele was somatically methylated in 81% of cases.
Demethylation studies using 5'-aza 2'-deoxycytidine have demonstrated re-
expression of OBCAM in a CpG island methylated, non-expressing clonal
derivative of the ovarian cancer cell line, SKOV3.
OBCAM transfection into this methylated SKOV3 clonal derivative resulted
in functional phenotypes in keeping with TSG function. OBCAM re-
expression resulted in suppressed growth and enhanced aggregation of cells
in vitro, markedly suppressed sub-cutaneous tumor growth in vivo and almost
completely abolished intra-peritoneal tumorigenicity.
OBCAM mutation analysis by SSCPE across approximately 200 ovarian
primary tumors and cell lines has identified a single somatic mis-sense
(Proline to Arginine) mutation within the first Ig domain in an ovarian tumor.
As somatic mutation is an infrequent event, this supports abrogation of
expression by LOH and by epigenetic mechanisms as the favoured means of
OBCAM inactivation in epithelial ovarian cancer.
The data presented is the first description of involvement of the IgLON
family in cancer.
5.6
REAL TIME QUANTITATIVE POLYMERASE CHAIN REACTION
ANALYSIS OF HPV 16 IN ADENOCARCINOMA OF CERVIX
GK Chew1
*, ME Cruickshank1
, PH Rooney2
, DE Parkin1
and GI Murray2
.
1
Department of Gynaecology-Oncology, Aberdeen Royal Infirmary,
Aberdeen.
2
Department of Pathology, University of Aberdeen, Aberdeen.
There has been a relative and absolute increase in the incidence of
adenocarcinoma of the cervix (ACC), particularly in the younger women.
ACC represents 15-20% of all cervical cancer and will become more
prominent as the incidence of squamous cell carcinoma of the cervix (SCC)
continues to fall with organised cervical screening. Both HPV 16 and 18 are
associated with ACC. In some in-situ polymerase chain reaction (PCR), HPV
16 was noted to be present in the adjacent uninvolved cervical epithelium. In
this study, we have coupled two powerful techniques, laser capture
microdissection and real-time quantitative PCR to determine the HPV16
prevalence in the women with ACC and to accurately assess the HPV16
DNA copy number in the cells. The association between HPV16 positivity
and the age of the women, grade and stage of the cancer was analysed.A
cohort of women diagnosed with ACC between 1991-2000 inclusive was
identified. The histology was reviewed to confirm the diagnosis and
adenosquamous carcinomas were excluded. The ACC cells were isolated
from archival paraffin-fixed 5m sections using a PixCell II laser
microdissection system (Arcturus Engineering). Tumour tissue was identified
and removed by the laser onto a transfer film. Approximately 200 laser pulses
were taken per tumour specimen. DNA extraction was carried out by
incubating the dissected cells in a 3mg/ml proteinase K at 55C for 4 hours
and then heat inactivated. DNA was assessed by Taqman PCR using the
ABI7700 (PE Applied Biosystems). Real time quantitative PCR was used to
amplify the internal control gene, Beta-globin and the HPV 16 gene. The
Caski cell line is known to have 600 copies HPV16 DNA per cell. DNA was
extracted from a known number of Caski cells and serially diluted to 10-9
concentration. Real-time quantitative PCR was used to determine the Ct count
for Beta-globin and HPV 16 at the different concentrations in order to
generate a standard curve. For each case, the HPV16 and Beta-globin gene
copy number was calculated using the mean values of Ct (each case was
assayed in triplicate) and plotted against the standard curve to determine the
gene copy number.55 women were identified to have ACC cervix from 1991-
2000 inclusive. The age distribution of this cohort showed a bimodal pattern,
with peaks in the 36-45 and >55 years age groups. In 3 cases, the DNA
extraction was unsuccessful and these cases were excluded. Of the remaining
52 women, 25% were HPV 16 positive. HPV positivity was more common in
the well and moderately differentiated tumours and in women less than 45
years old. There is no difference in the HPV copy number in the grade and
stage of the cancer. The HPV 16 gene copy number is significantly higher in
women more than 45 years old (p=0.03).
Acknowledgements: This research was funded by Grampian University
Hospitals NHS Trust Endowments, Gynaecology Oncology Research Fund
and University of Aberdeen development trust.
Oral Presentations
S24
British Journal of Cancer (2002) 86(Suppl 1), S13 - S33  2002 Cancer Research UK
Oral Presentations
S25
 2002 Cancer Research UK British Journal of Cancer (2002) 86(Suppl 1), S13 - S33
6.2
BEXXAR


: OVER FIVE YEARS EXPERIENCE IN TWO UNITED
KINGDOM CENTRES
A.J.Davies1
(*), J.A.Radford2
, A.Z.S.Rohatiner1
, K.Britton1
, S.Owens2
,
D.P.Deakin2
, S.Howell2
, B.M.Carrington2
, J.A.Lawrance2
, I.N.Micallef1
,
S.Vinnicombe1
, J.Clayton2
, J.Matthews1
, M.Harris2
, A.Norton1
and
T.A.Lister1
.
1
St Bartholomew's Hospital, London. 2
Christie Hospital, Manchester, UK.
Between March 1996 and March 2001, 96 patients (pts.), aged 27-90 (median
53 years) with recurrent or refractory follicular lymphoma (FL) 64 pts.,
mantle cell lymphoma 5 pts., lymphoplasmacytic lymphoma (LPC) 4 pts.,
small lymphocytic lymphoma 3 pts. and a further 20 pts. who had undergone
transformation (Tx) to large B cell lymphoma (LBCL, 19 pts. previous FL, 1
pt. LPC) were enrolled to be treated either in an open phase II trial (61 pts.)
or on a compassionate basis (35 pts.) with Bexxar(tositumomab and I131
tositumomab). Forty patients had bone marrow infiltration (all <25%). The
median number of previous therapies was 2 (range 1-9). Eleven pts. had
progressed following previous high-dose therapy (HDT), Rituximab (RXB)
13pts., or Bexxar 3 pts. Administration, after dosimetry, was as previously
described, delivering a whole body dose of 75cGy if the platelet (plt.) count
was >150x109
/l, 65 cGy for pts. with a plt. count of 100-149x109
/l and 45cGy
following HDT. Seven pts. did not receive therapy; 3 because of human anti-
mouse antibodies, 1 pt. withdrew and 3 progressed after dosimetry. Response
was first evaluated 7 weeks post therapy and repeated 3 monthly. Further
disease regressions were observed for upto 1 year, although progressions
occurred in other pts. during this time. Toxicity was principally
haematological. Median neutrophil and plt. nadirs occured at 7 and 6 weeks
respectively. Grade 3 or 4 toxicity: neutropenia in 38% of pts. and
thrombocytopenia in 31%. With a median follow up of 21
/4 years, the median
duration of response was 21
/4 years (95% CI:1.1 to not reached) for those in
CR/CR(u) and 7 months (3 mo. to not reached) for those in PR. Overall
response rate at first evaluation declined with successive number of previous
therapies: 73%, 66% and 46% respectively for 1, 2 or 3 or more treatments.
The efficacy of Bexxar has been demonstrated in a range of clinical
settings with the longest remission duration in those who achieve CR/CR(u).
Supported by the ICRF, CRC, Corixa Corp and the NHS.
At 7 weeks ORR At 7 weeks
CR/CR PR
At 7 Weeks TotalReaching
CR/CR(u)
All patients 59% (55/93) 10% (9/93) 49% (46/93) 25%(23/93)
FL 70% (44/63) 13% (8/63) 57% (36/63) 30% (19/63)
Tx to LBCL 32% (6/19) 5% (1/19) 26% (5/19) 16% (3/19)
Other Histology 36% (4/11) 0% (0/11) 36% (4/11) 0% (0/11)
Previous RXB 62% (8/13) 0%(0/13) 62% (8/13)
Previous HDT 36% (4/11) 9% (1/11) 27% (3/11)
(Responses based upon total no. of pts. that received dosimetry. 2 additional pts. are non
evaluable.
6.3
A PHASE I/II DENDRITIC CELL (DC) IMMUNOTHERAPY TRIAL
FOR HEPATOCELLULAR CARCINOMA (HC)
*Rachel S Midgley1
, David J Kerr1
, Noweeda Mirza2
, Daniel Palmer2
, David
Adams2
, Lawrence Young2
1
ICRF Medical Oncology Unit. Churchill Hospital. Oxford
2
CRC Institute for Cancer Studies, University of Birmingham
Background and Purpose: Cytotoxic tumour infiltrating lymphocytes (TILs)
are observed in HC, a tumour with poor prognosis and no established
treatment. This phase I/II trial was designed to assess the safety and efficacy
of dendritic cell vaccination, administered in an attempt to convert latent anti-
tumour immunological responses into effective tumour rejection.
Patients and Methods: 21 patients (16M, 5F) with surgically non-curative HC
(2 post-optimal debulking; 5 post-chemotherapy) have been recruited. DCs
are prepared from peripheral blood by culture of plastic-adherent peripheral
blood mononuclear cells (PBMCs) in GM-CSF and IL4 (phenotype
confirmed by FACS analysis). TNF--matured DCs (3-5x106
) are pulsed
with hepatoma (hepG2)-lysate, washed and re-injected intravenously.
Patients undergo vaccination every 3 weeks and, if achieving stable or
responding disease ( bi-dimensional measurement on CT or US), after 3
cycles, a further 3 vaccinations are offered. Immunological assays (T-cell
proliferation, -IFN ELIspot) of patient non-adherent cell populations (T-cell
enriched) are performed with each cycle.
Results: 65 DC vaccinations have been administered with 12 patients
assessable for response and toxicity, 5 assessable for toxicity alone, 1 non-
assessable, and 3 have not yet reached the review point. Of 12 assessable for
response - 1 partial response (confirmed), 3 disease stabilisations, and 8
progressive disease. Toxicity was mild with G2 nausea/vomiting (n=2), G1
flu-like illness (n=3), G1 fever (n=2).
Conclusion: This DC vaccination study demonstrates low toxicity and some
clinical activity that warrants further patient recruitment. Parallel
immunological assays will be presented. Comparison of intravenous and sub-
cutaneous administration is now underway.
All work was governed by standards for Good Clinical Practice and reviewed
regularly by the Local Research Ethics Committee.
6.4
THALIDOMIDE ANALOGUE CDC-501 IS SAFE AND WELL
TOLERATED BY PATIENTS WITH END STAGE CANCER AND
SHOWS EVIDENCE OF CLINICAL RESPONSES AND EXTENSIVE
IMMUNE ACTIVATION
JB Marriott1
, IA. Clarke1
, K Dredge1
, H Pandha1
, H Kristaleit1
, A
Polychronis1
, GW Muller2
, D Stirling2
and AG. Dalgleish1
Division of Oncology, St George's Hospital Medical School, Cranmer
Terrace, London, SW17 ORE, UK. Celgene Corporation, Warren, NJ, USA.
PURPOSE: To assess the safety, tolerability, efficacy, immune stimulatory
and anti-angiogenic effects of a novel thalidomide analogue, CDC-501
(REVIMID), in the treatment of patients with advanced cancer. PATIENTS
AND METHODS: Twenty patients with heavily pre-treated advanced stage
IV malignant melanoma (n=13), pancreatic (n=2) and other cancers (n=5)
were treated with a dose escalating regimen of CDC-501 starting at 5mg and
rising to 50mg after 4 weeks. Clinical efficacy, tolerance and adverse effects
were evaluated. In vitro analysis of peripheral T cell surface markers and
serum for cytokines and pro-angiogenic factors were performed. RESULTS:
CDC-501 was generally well tolerated and no serious adverse events were
attributed to its use. Fourteen patients completed the study, 3 withdrew due to
disease progression and 3 due to withdrawal of consent. CDC-501 treatment
led to clinical responses in a number of patients. All patients showed
evidence of activation of both CD4+ and CD8+ T cells as measured by
increased CD45RO+ expression. Furthermore, activation of memory cells
(CD45RA+/L-selectinlow) in some patients may indicate activation of tumour-
specific cells. This was associated with increased serum levels of GM-CSF,
sIL-2R, IL-12 and TNF-. However, levels of pro-angiogenic factors were
unchanged. CONCLUSION: This study demonstrates the safety and
significant clinical efficacy of CDC-501 in the treatment of recalcitrant
advanced cancer. The induction of immune activation suggests a more
beneficial role for this class of compound in less advanced patients as an
immunostimulatory adjuvant perhaps combined with tumour cell vaccination
regimens.
6.5
PHASE 1 TRIAL OF HERPES SIMPLEX VIRUS, HSV1716
FOLLOWING INJECTION INTO BRAIN ADJACENT TO TUMOUR
IN PATIENTS WITH PRIMARY MALIGNANT GLIOMA.
Harrow, S.*, Papanastassiou, V., Brown, S.M., Fraser, M., Rampling, R.1
1
The University of Glasgow.
The most common primary brain tumours are glial tumours, including the
highly malignant glioblastoma. With current treatments of surgery,
radiotherapy and chemotherapy outcome remains poor with a median
survival of one year. A novel strategy to combat this disease is the utilisation
of the oncolytic herpes simplex virus, HSV1716. HSV1716 selectively
replicates in actively dividing cells.
Two phase I clinical trials using HSV1716 have been completed. In the first,
patients with recurrent glioma received direct intratumoural injection of
escalating virus dose. In the second, patients with recurrent as well as newly
diagnosed high grade glioma were injected with HSV1716 into the tumour
prior to resection at 5-8 days. No toxicity was seen in any patients and
Oral Presentations
S26
British Journal of Cancer (2002) 86(Suppl 1), S13 - S33  2002 Cancer Research UK

				

